# 

**Published:** 1882-03

**Authors:** 


### CONTENTS:

ART. I.—Fillings. By J. M. Hurtt, D. D. S.......481
ART. II.—A Generalized Treatment of Irregularities. By Walter H. Coffin, F. C. S.......495
ART. III.—Suggestions Relating to the Causes of Rapid Dental Decay. By C. W. Spalding, D. D. S.......504
ART. IV—The Denuding of Teeth. By Geo. W. McElhaney, D. D. S.......512
ART. V.—Cause of Death Under Chloroform. By Dr. G. F. Hackenberg,.......515
Dental Dealers' Convention.......517
Georgia State Dental Society.......518

### EDITORIAL, ETC.

The Forty-Second Annual Commencement of the Baltimore College of Dental Surgery.......518
Vanderbilt University, Department of Dentistry.......521
Correspondence—Stannous Gold.......521

### OBITUARY.

Dr. M. S. Dean.......522

### MONTHLY SUMMARY.

A Tooth in the Bronchus Causing Pneumonia.......522
Annealing Gold.......525
An Anæsthetic Car.......525
Keeping Warm.......526
Nerve Regeneration.......528

